# Alteration of Skin Microbiome in CKD Patients Is Associated With Pruritus and Renal Function

**DOI:** 10.3389/fcimb.2022.923581

**Published:** 2022-06-28

**Authors:** Yu Tian, Chaoqun Gu, Feng Yan, Yifeng Gu, Yangkun Feng, Jie Chen, Jiayi Sheng, Lei Hu, Peng Jiang, Wei Guo, Ninghan Feng

**Affiliations:** ^1^Department of Urology, Wuxi No. 2 People’s Hospital, Affiliated Wuxi Clinical College of Nantong University, Wuxi, China; ^2^Department of Nephrology, Wuxi No. 2 People’s Hospital, Affiliated Wuxi Clinical College of Nantong University, Wuxi, China; ^3^Collaborative Innovation Center for Diagnosis and Treatment of Infectious Diseases, State Key Laboratory for Diagnosis and Treatment of Infectious Diseases, The First Affiliated Hospital, School of Medicine, Zhejiang University, Hangzhou, China; ^4^School of Medicine, Nantong University, Nantong, China

**Keywords:** chronic kidney disease, *Escherichia–Shigella*, *Oribacterium*, pruritus, renal function

## Abstract

Dysbiotic gut microbiome in chronic kidney disease (CKD) patients has been extensively explored in recent years. Skin microbiome plays a crucial role in patients with skin diseases or even systemic disorders. Pruritus is caused by the retention of uremic solutes in the skin. Until now, no studies have investigated the role of skin microbiome in CKD and its association with pruritus. Here, we aim to examine the bacterial profile of skin microbiome in CKD and whether it is correlated to pruritus. A total of 105 CKD patients and 38 healthy controls (HC) were recruited. Skin swab was used to collect skin samples at the antecubital fossa of participants. Bacterial 16S rRNA genes V3–V4 region was sequenced on NovaSeq platform. On the day of skin sample collection, renal function was assessed, and numeric rating scale was used to measure pruritus severity. Principal coordinate analysis (PCoA) revealed a significant difference in bacterial composition between the groups of CKD and HC. A depletion of bacterial diversity was observed in CKD patients. *Akkermansia*, *Albimonas*, *Escherichia–Shigella*, etc. showed significant higher abundance in CKD patients, whereas *Flavobacterium*, *Blastomonas*, *Lautropia*, etc. significantly declined in patients. *Escherichia–Shigella* achieved an acceptable diagnostic biomarker with area under the curve (AUC) value of 0.784 in the receiver operating characteristics (ROC) curve. In addition, CKD patients with pruritus (P-CKD) had a different bacterial community comparing to those without pruritus (non-P-CKD) and HC group. Several bacterial genera showing significant difference between P-CKD and non-P-CKD/HC, such as *Oribacterium*, significantly declined in P-CKD patients than that in the HC group, and *Methylophaga* significantly increased in P-CKD patients compared to that in HC subjects. *Escherichia–Shigella* was positively associated with the levels of pruritus severity, blood urea nitrogen (BUN), uric acid, and urine protein; *Oribacterium* was negatively associated with pruritus severity, whereas it was positively associated with estimated glomerular filtration rate (eGFR) and 24-h urine volume. The dysbiotic of skin microbiome in CKD patients and its association with pruritus and renal function shed a light on skin probiotics.

## Introduction

Chronic kidney disease (CKD) is an important contributor to morbidity and mortality from non-communicable diseases. In 2017, 697.5 million cases of all-stage CKD were recorded, for a global prevalence of 9.1% (Collaboration GCKD, 2020). CKD progression to end-stage renal disease often requires an expensive renal replacement therapy, such as hemodialysis, peritoneal dialysis, or kidney transplantation. As many as 9 in 10 adults with CKD do not know they have CKD, and about two in five adults with severe CKD do not know they have CKD (Prevention CFDC, 2021); it is important to investigate diagnostic biomarkers and therapies for CKD.

Gut microbiome has gained attention and is increasingly noted to play a significant role in a number of disease states, including CKD. Several previous studies demonstrated that CKD patients can be characterized with lower bacterial richness and diversity (Hu et al., 2020; Ren et al., 2020; Liu et al., 2021). Several bacterial genera in the gut can be considered biomarkers for CKD patients. For example, Wang et al. demonstrated that CKD patients had increased abundance of *Eggerthella lenta*, *Flavonifractor* spp., *Alistipes* spp., *Ruminococcus* spp., and *Fusobacterium* spp (Wang et al., 2020). Ren et al. reported that *Klebsiella* and Enterobacteriaceae increased in CKD group compared to that in HC subjects (Ren et al., 2020). We previously found that *Bifidobacterium* and *Bifidobacterium longum* predicted the prevalence of CKD (Liu et al., 2021).

Like the gut, human skin has its own microbiome. Skin microbiome has important roles in educating the innate and adaptive arms of the cutaneous immune system. Disruption of skin microbiome is associated with skin disease or even systemic disease (Meijers et al., 2019; Huang et al., 2020). The skin in atopic dermatitis patients had a depletion of bacterial diversity and an elevation of *Staphylococcus aureus* and *Staphylococcus epidermidis* (Bjerre et al., 2017). Patients with systemic lupus erythematosus exhibited disordered skin microbiome accompanied with increased abundances of *Staphylococcus* and *Corynebacterium1* and a decreased abundance of *Cutibacterium*. However, no studies have reported the association between skin microbiome and CKD.

CKD-associated pruritus is defined as itching directly related to kidney disease, without another comorbid condition to explain itching. Pruritus is a distressing comorbidity found commonly in patients with end-stage renal disease, chronic kidney disease (CKD), and those on dialysis (Puneet et al., 2021). Pruritus has a major clinical impact, being associated strongly with poor quality of life, impaired sleep, depression, and increased mortality (Ahdoot et al., 2022). Although the pathogenesis of pruritus remains largely unclear (Combs et al., 2015), it is demonstrated that pruritus is mainly ascribed to the retention of uremic solutes (Kim et al., 2021).

Skin microbiome is correlated with health state, and the accumulation of uremic solutes in the skin contributes to pruritus in CKD patients. Here, we hypothesized that CKD patients had a dysbiotic skin microbiome, and it played a role in pruritus severity.

## Methods and Materials

### Subject Recruitment

The ethics committee of the Affiliated Wuxi Second Hospital of Nanjing Medical University approved this study (Ref. 2018051). Informed consent was provided by all subjects prior to sample collection. Adult CKD patients and healthy controls (HC) were recruited. Diagnostic criteria of CKD include a decreased estimated glomerular filtration rate (eGFR) [<60 ml/min/1.73m^2^ or evidence of kidney damage such as albuminuria (albumin excretion rate ≥30 mg/24 h; urinary albumin creatinine ratio (UACR) ≥30 mg/g], urine sediment abnormalities, electrolyte and other abnormalities due to tubular disorders, abnormalities detected by histology, structural abnormalities detected by imaging, or history of kidney transplantation (Perez-Gomez et al., 2019). CKD patients undergoing hemodialysis were excluded from the study. The exclusion criteria for HC were as follows: those with the following illness or health conditions, namely, kidney damage; positive urinary protein or eGFR <90 ml/min/1.73m^2^; diabetes; hypertension; cancer; acute intercurrent disease and infections; diarrhea; kidney transplantation; pregnancy; breastfeeding; allergic history; those who used antibiotics, probiotics, or immunosuppressive drugs; and ultraviolet therapy within the past 30 days before enrollment. In addition, current or those had a history of pruritus were excluded from the HC group, and subjects with allergic history were excluded from both the CKD and HC group.

CKD staging criteria is as follows: Stage 1, kidney damage with normal eGFR (<90 ml/min); Stage 2, mild reduction in eGFR (60–89 ml/min); Stage 3a, moderate reduction in eGFR (45–59 ml/min); Stage 3b, moderate reduction in eGFR (30–44 ml/min); Stage 4, severe reduction in eGFR (15–29 ml/min); and Stage 5, renal failure (eGFR <15 ml/min) (Stevens and Levin, 2013).

### Clinical Data Collection

Current illness, such as diabetes, hypertension, and cancer, was assessed by reviewing clinical records and medical interviews. The renal function was examined on the day of sample collection. Numeric rating scale (NRS) was used to measure itching severity in participants (Phan et al., 2012). The patients were asked to record their current pruritus intensity on a NRS from 0 (“no itch”) to 10 (“worst imaginable itch”) on a questionnaire (Erickson and Kim, 2019).

### Sample Collection and DNA Isolation

Participants were asked to avoid bathing/showering and application of any topical agents on their left hand 24 h prior to the sample collection. A 4-cm^2^ area from the antecubital fossa was firmly swabbed for at least 30 s (Grice et al., 2008). The swab was immediately placed in a sterile container, which has been added with 1 ml sterile ddH_2_O before sample collection. All samples were immediately stored at −80°C until further processing. DNeasy PowerSoil Pro Kit was used to isolate microbial genomic DNA from samples (Hilden, Qiagen, Germany), and the isolation procedures were performed according to the manufacturer’s instructions. The total DNA was eluted in 50 μl of elution buffer and stored at −80°C until used for PCR.

### 16S rRNA Sequencing

PCR products were confirmed by 2% agarose gel electrophoresis. Throughout the DNA extraction process, ultrapure water, instead of a sample solution, was used as a negative control to exclude the possibility of false-positive PCR results. Polymerase chain reaction (PCR) amplification of the bacterial 16S rRNA genes V3–V4 region was performed using the universal primers 338F and 806R with 30 cycles. The PCR products were purified with AMPure XT beads (Beckman Coulter Genomics, Danvers, MA, USA) and quantified by Qubit (Invitrogen, Waltham, MA, USA). Amplicon pools were prepared for sequencing, and the size and quantity of the amplicon library were assessed using Agilent 2100 Bioanalyzer (Agilent, Santa Clara, MA, USA) and Library Quantification Kit for Illumina (Kapa Biosciences, Woburn, MA, USA), respectively. The libraries were sequenced using the NovaSeq PE250 platform. To avoid any impact of batch effects, we processed and sequenced all of the saliva samples at once.

### Bioinformatic Analysis

Paired-end reads were assigned to samples based on their unique barcodes and truncated by cutting off the barcode and primer sequence. Paired-end reads were merged using FLASH. Quality filtering of the raw reads was performed under specific filtering conditions to obtain high-quality clean tags according to fqtrim (v. 0.94). Chimeric sequences were filtered using Vsearch software (v. 2.3.4). After dereplication using DADA 2, we obtained a feature table and feature sequence. Alpha diversity and beta diversity were calculated by normalization to the same sequences randomly using QIIME2. Next, according to the SILVA (v. 132) classifier (Phan et al., 2012), feature abundance was normalized using the relative abundance of each sample. Alpha diversity was applied to analyze the complexity of species diversity for a sample through the Chao1, Shannon, and Simpson indices. Beta diversity analysis was performed to evaluate differences in ASVs between samples, and permutational multivariate analysis of the variance method was used to examine feature differences between groups of CKD and HC based on unweighted unifrac distance; statistical significance was defined as *p* < 0.05. The sequencing data obtained in this study have been deposited in GenBank Sequence Read Archive under accession number SRP370056 (https://www.ncbi.nlm.nih.gov/bioproject/PRJNA825583).

### Statistical Methods

Descriptive statistics for demographics and clinical characteristics of the CKD and HC groups are presented. To compare demographics and clinical characteristics between the CKD and HC groups, continuous variables were assessed using independent *t*-tests and categorical variables using chi-square or Fisher’s exact tests, when appropriate.

R software (version 3.6.3) was used to perform bioinformatic analysis. The Wilcoxon rank sum test was applied to compare bacterial diversity and the relative abundance of bacterial taxa and metabolic pathways between the CKD and HC groups, and a Benjamini Hochberg false discovery rate (FDR)-corrected *p*-value was calculated for comparative tests. A *p* < 0.05 was used as a cutoff for comparative statistical tests. Next, the bacterial genera displaying significant difference between CKD and HC were selected to perform the correlation analysis between bacterial genus and pruritus severity and renal function.

## Results

### Demographics

A total of 103 CKD patients and 38 HC were recruited from December 2018 to November 2020. As expected, compared to the HC group, the CKD patients had significantly higher eGFR, serum urea, serum creatinine, and serum uric acid (*p* < 0.05; (**Table 1**). There were 77 CKD patients with pruritus (P-CKD) and 26 patients without pruritus (non-P-CKD). The P-CKD patients had significantly higher age, serum urea, serum creatinine, urine protein, and urine albumin/creatinine ratio comparing to non-P-CKD patients (p<0.05; **Supplementary Table 1**).

**Table 1:** Characteristics of the participants.

Parameters	CKD (n = 103)	HC (n = 38)	*P*-value
Age, year	59.32 ± 16.79	56.50 ± 16.98	0.379
Men	56 (54.37)	17 (44.74)	0.607
Body mass index (kg/m^2^)	25.43 ± 5.54	23.85 ± 2.56	0.106
eGFR (ml/min/1.73 m^2^)	57.59 ± 35.76	117.45 ± 45.57	<0.001
Serum urea (mmol/L)	11.90 ± 10.38	4.99 ± 1.42	<0.001
Serum creatinine (mg/dl)	203.79 ± 194.41	59.85 ± 10.81	<0.001
Serum uric acid	397.91 ± 137.81	265.41 ± 75.50	<0.001
Urine protein	1.11 ± 1.42	1.22 ± 1.93	0.812
24-h urine protein	2,940.42 ± 2,234.96	NA	NA
24-h urine volume	1,869.44 ± 631.05	NA	NA
Urine microalbumin	178.52 ± 55.97	NA	NA
Urine albumin/creatine ratio	38.11 ± 15.48	NA	NA
CKD stage			
Stage 1	18 (17.48)	NA	NA
Stage 2	33 (32.04)	NA	NA
Stage 3	22 (21.36)	NA	NA
Stage 4	12 (11.65)	NA	NA
Stage 5	18 (17.48)	NA	NA
Co-current disease			
Hypertension	76 (73.79)	NA	NA
Type 2 diabetes mellitus	28 (27.18)	NA	NA

Pearson’s Chi-square/Fisher’s exact test was used to compare dichotomous variables, and an independent t-test was used to compare continuous variables.

### CKD Patients Had a Distinct Bacterial Community

In total, 11,803,437 raw reads were obtained (average raw reads were 83,712; ranging from 53,514 to 87,895); 10,988,455 reads were obtained after removing low-quality or ambiguous reads. Good coverage ranged from 98.81% to 100.00%.

**Figure 1:**
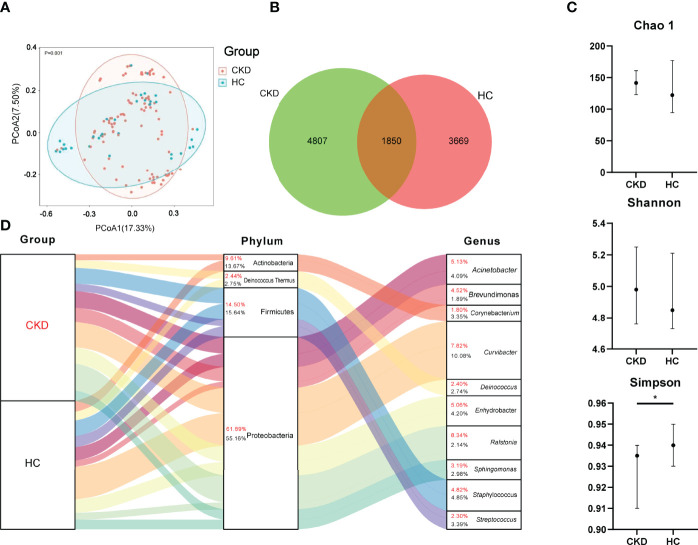
Bacterial community, Venn, diversity, and composition in the groups of CKD and HC. **(A)** PCoA based on Bray–Curtis distances at ASV level showed different microbial compositions between groups of CKD patients and HC (*p* < 0.05). Permutational multivariate analysis of variance (PERMANOVA) was performed for statistical comparisons of samples in the two cohorts. *p*-value was adjusted by the Benjamini and Hochberg false discovery rate (FDR). **(B)** Venn diagram showing a dissimilar number of ASVs shared by the groups of CKD and HC. **(C)** Bacterial richness and diversity measured by Chao1, Shannon, and Simpson were calculated at the microbial ASV level. Wilcoxon rank-sum test was performed and adjusted by Benjamini and Hochberg false discovery rate (FDR). ^*^p < 0.05. **(D)** Bacterial profile at the phylum and genus level. Sankey plot representing overall gut mycobiome composition and corresponding abundance area for CKD patients and HCs. The taxonomic classification levels of phylum and genus are displayed. The top 10 most abundant genera and their affiliated phyla are shown in the Sankey plot.

Principal coordinate analysis (PCoA) revealed a significant difference in bacterial composition between the CKD and HC groups (*p* = 0.001; **Figure 1A**). The Venn diagram in **Figure 1B** illustrates 6,657 and 5,519 observed features in the CKD and HC samples, respectively, of which 1,850 (15.19%) were shared. Based on Wilcoxon rank analysis, only bacterial diversity index of Simpson was significantly decreased in the CKD group compared to the HC group (*p* < 0.05; **Figure 1C**).

### Bacterial Profile Comparison Between Groups of CKD and HC

As shown in **Figure 1D**, Proteobacteria, Firmicutes, Deinococcus Thermus, and Actinobacteria were the most abundant bacterial phyla in the participants. When the bacterial phylum was compared between the groups of CKD and HC, Actinobacteria, Bacteroidetes, and Epsilonbacteraeota were significantly enriched in the group of CKD (*p* < 0.05; **Supplementary Figure 1**).

When the bacterial genus level was assessed, *Ralstonia* (8.34%), *Curvibacter* (7.82%), *Acinetobacter* (5.13%), *Enhydrobacter* (5.06%), and *Staphylococcus* (4.82%) were dominated in CKD patients (**Figure 1D**). The predominated bacterial genus in the HC group were *Curvibacter*, *Staphylococcus*, *Enhydrobacter*, *Acinetobacter*, and *Streptococcus*, which accounted for 10.08%, 4.85%, 4.20%, 4.09%, and 3.39%, respectively (**Figure 1D**).

**Figure 2:**
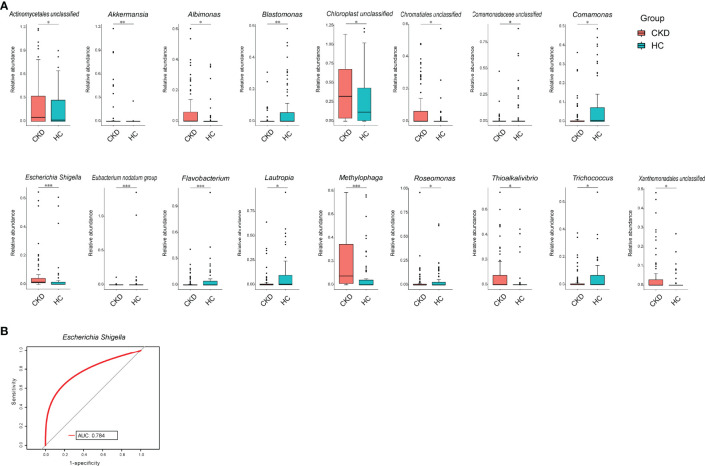
Bacterial comparison between CKD and HC. **(A)** Bacterial genera that were differentially abundant between CKD patients and HCs. Only the genera with above 1‰ are displayed. *p*-value was calculated using Wilcoxon rank-sum test and adjusted by Benjamini and Hochberg false discovery rate (FDR). *p < 0.05, **p < 0.01, and ***p < 0.001. **(B)** Receiver operating characteristic curve (ROC) curve for validation of bacterial classification of CKD from HC.

We compared the bacterial genus with relative abundance ≥1‰ between the groups of CKD and HC. *Akkermansia*, *Albimonas*, *Escherichia–Shigella*, *Methylophaga*, and *Thioalkalivibrio* showed significant higher abundance in CKD patients. In addition, unclassified bacterial genera in Actinomycetales, Chloroplast, Chromatiales, and Xanthomonadales were also enriched in CKD patients (*p* < 0.05; **Figure 2A**), whereas in the Eubacterium nodatum group, *Flavobacterium*, *Blastomonas*, *Lautropia*, *Exiguobacterium*, *Roseomonas*, *Comamonas*, *Trichococcus*, and an unclassified bacterial genus in Comamonadaceae significantly declined in CKD patients (*p* < 0.05; **Figure 2A**). To look for potential biomarkers that could distinguish CKD patients from HC, receiver operating characteristics (ROC) curve was obtained. From this analysis, *Escherichia–Shigella* achieved an area under the curve (AUC) value of 0.784 (**Figure 2B**), indicating that it can be considered as an acceptable diagnostic biomarker for CKD (Mandrekar, 2010).

### Alteration of Bacterial Community in CKD Patients in Various Stages

**Figure 3:**
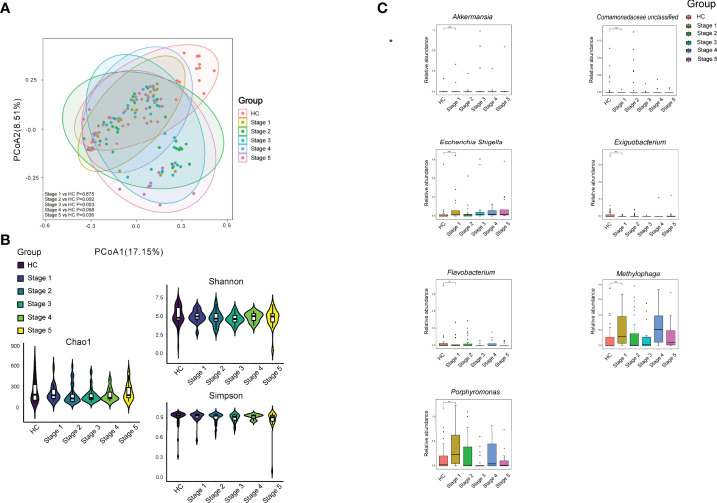
Bacterial community, diversity, and genus comparison in the subgroups according to CKD stages and HC. **(A)** PCoA based on Bray–Curtis distances at ASV level showed different microbial compositions between subgroups of CKD stage and HC. Permutational multivariate analysis of variance (PERMANOVA) was performed for statistical comparisons of samples in the six groups. *p*-value was adjusted by the Benjamini and Hochberg false discovery rate (FDR). **(B)** Bacterial richness and diversity measured by Chao1, Shannon, and Simpson were calculated at the microbial ASV level. Wilcoxon rank-sum test was performed and adjusted by Benjamini and Hochberg false discovery rate (FDR). **(C)** Comparison of the abundances of bacterial genus in CKD patients in each stage and HC. *p < 0.05, **p < 0.01, and ***p < 0.001, respectively.

When the patients were separated into subgroups based on CKD stage, significant differences between the patients in various CKD stages and HC were observed, except for Stages 1 and 4 (*p* < 0.05; **Figure 3A**). However, we noticed that there was no significant difference between patients in various CKD stages, such CKD stage 1 vs. CKD stage 2 (*p* > 0.05). In addition, the bacterial richness and diversity did not show significant difference between HC and patients in each CKD stage and among CKD stages (*p* > 0.05; **Figure 3B**). We compared the bacterial genus with ≥1‰ relative abundance between HC and CKD stages and among the five stages and found that CKD patients in Stage 1 had significantly higher levels of *Akkermansia*, *Escherichia–Shigella*, *Methylophaga*, and *Porphyromonas*, while *Exiguobacterium*, *Flavobacterium*, and a bacterial genus in Comamonadaceae significantly declined in Stage 1 CKD patients compared to that in HC (*p* < 0.05; **Figure 3**).

### CKD Patients With Pruritus had a Distinct Bacterial Community

**Figure 4:**
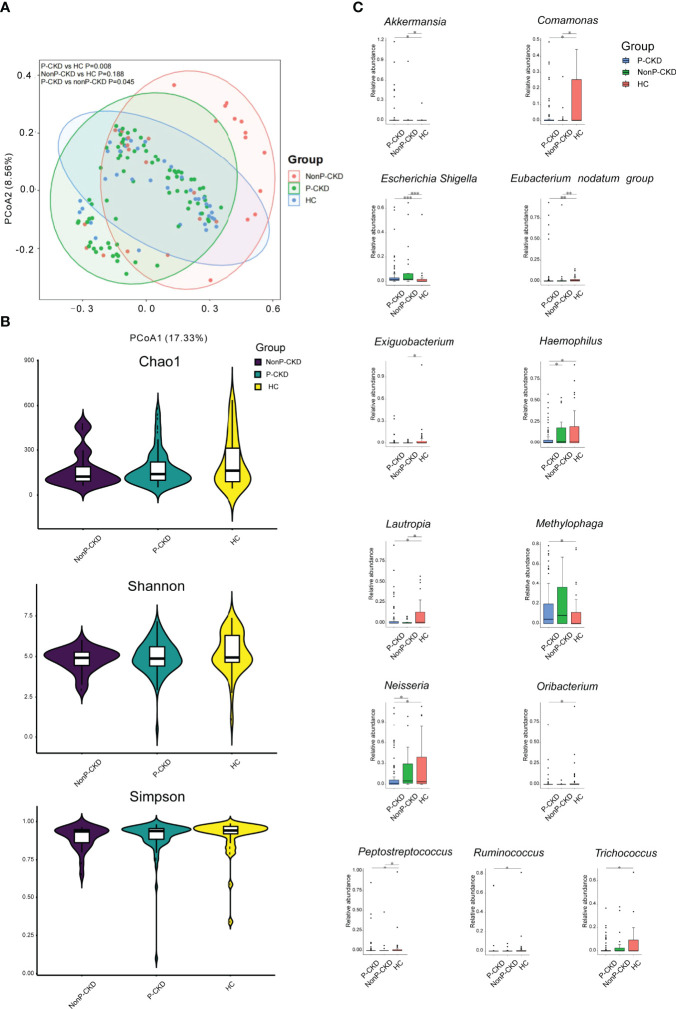
Bacterial community, diversity, and genus comparison in the groups P-CKD, nonP-CKD, and HC. **(A)** PCoA based on Bray–Curtis distances at ASV level showed different microbial compositions among P-CKD, non-P-CKD, and HC. Permutational multivariate analysis of variance (PERMANOVA) was performed for statistical comparisons of samples in the three groups. *p*-value was adjusted by the Benjamini and Hochberg false discovery rate (FDR). **(B)** Bacterial richness and diversity measured by Chao1, Shannon, and Simpson were calculated at the microbial ASV level. Wilcoxon rank-sum test was performed and adjusted by Benjamini and Hochberg false discovery rate (FDR). **(C)** Comparison of the abundances of bacterial genus in P-CKD, non-P-CKD, and HC. Wilcoxon rank-sum test was performed and adjusted by Benjamini and Hochberg false discovery rate (FDR). ^*^p < 0.05, **p < 0.01, and ***p < 0.001.

When we compared P-CKD patients with non-P-CKD patients and HC, we found that P-CKD group had a different bacterial community from the groups of HC and non-P-CKD (*p* < 0.05; **Figure 4A**). However, the bacterial richness and diversity did not show difference among the three groups (*p* > 0.05; **Figure 4B**). When the bacterial genus with relative abundance ≥1‰ were compared among the three groups, *Haemophilus* showed significant decline in P-CKD patients than that in non-P-CKD and HC group. Meanwhile, *Oribacterium* significantly declined in P-CKD patients than that in HC group, and *Methylophaga* significantly increased in P-CKD patients compared to that in HC subjects (*p* < 0.05; **Figure 4C**).

### The Association of Renal Function and Pruritus Severity With Bacterial Community

**Figure 5:**
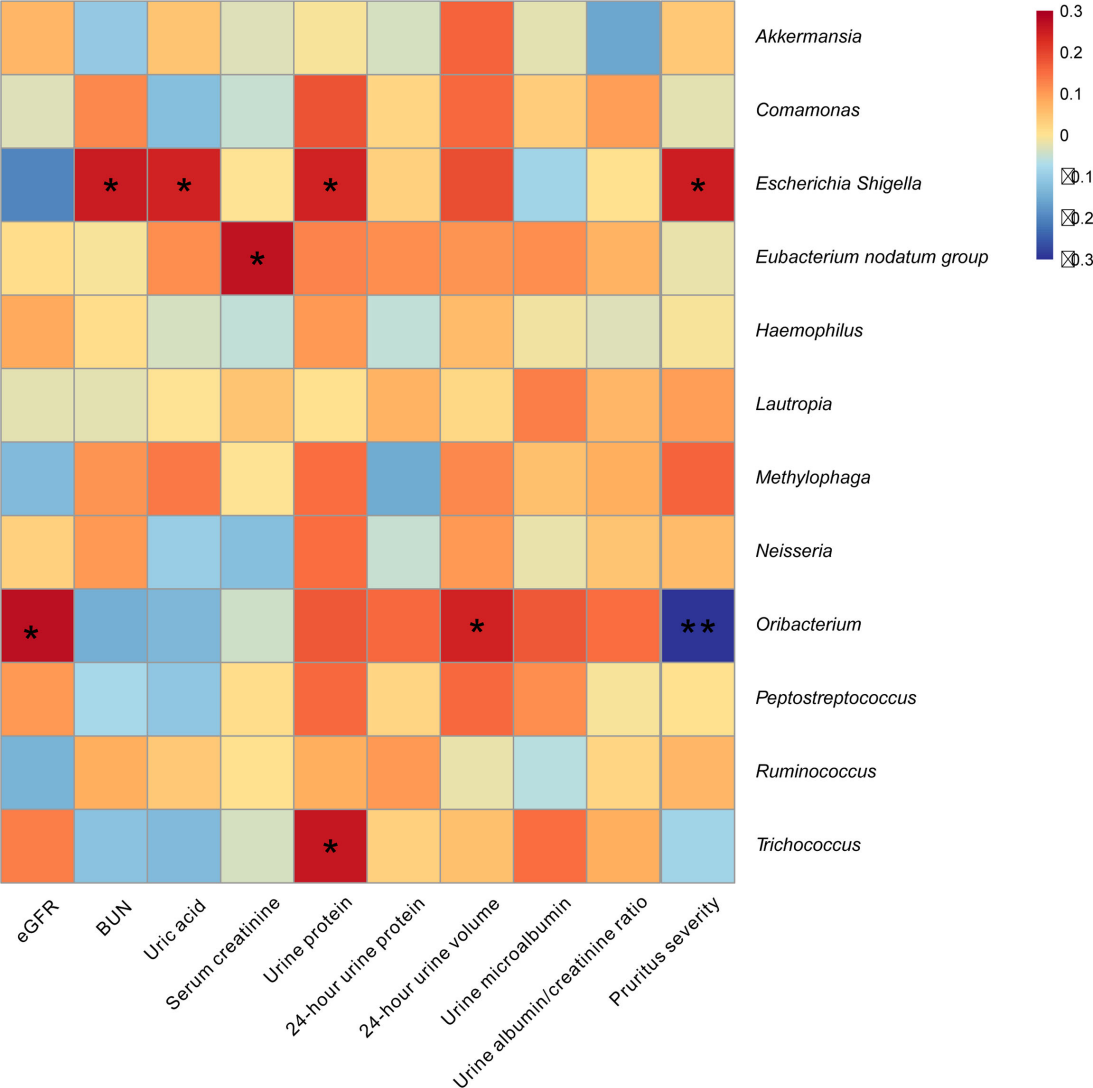
Skin microbiome was associated with pruritus and renal function in CKD patients. The heatmap depicted the association between the taxa which differed in CKD relative to HC and the value of pruritus and renal function in CKD patients. Spearman correlation analysis was performed. The correlation of two variables with values of |r| > 0.3 and *p <* 0.05 are displayed. ^*^p < 0.05 and **p < 0.01.

To assess the influence bacterial microbiome on renal function and pruritus severity in CKD patients, we performed Pearson analysis using the bacterial genus, which showed significant difference between the CKD and HC group. We found that *Escherichia–Shigella* was positively associated with the levels of blood urea nitrogen (BUN), uric acid, urine protein, and pruritus severity; *Eubacterium nodatum* group was positively associated with serum creatinine; *Trichococcus* was positively associated with urine protein; *Oribacterium* was negatively associated with pruritus severity, whereas it was positively associated with eGFR and 24-h urine volume (*p* < 0.05; **Figure 5**).

## Discussion

Most of the previous studies on skin microbiome were focused on skin disease, such as acne vulgaris, atopic dermatitis, and cutaneous squamous cell carcinoma (Francuzik et al., 2018; Mandrekar, 2010; O'Neill and Gallo, 2018; Wood et al., 2018). Here, we demonstrated that the profile of skin microbiome in CKD patients significantly differed from HC. It also showed that the microbial profile correlated substantially with the pruritus severity and renal function profiles in CKD patients.

First, we noticed that a distinct bacterial community was associated with CKD patients, which was demonstrated by both PCoA and Venn. The dysbiotic skin microbial community in CKD has never been reported by studies on human and animal models. The CKD patients exhibited lower bacterial diversity compared to HC group, which was consistent with bacterial gut microbiome in CKD patients (Hu et al., 2020; Ren et al., 2020; Liu et al., 2021). Although the bacterial community differed between CKD patients and HC group, the bacterial community was fluctuated with eGFR. We noticed that only CKD patients in Stages 2, 3, and 5 showed significant difference from HC group. Similar findings were found in our previous study on the gut of CKD patients (Liu et al., 2021). Additionally, our present study found that there was a difference in microbial community between the patients with pruritus and those without pruritus and healthy subject, indicating that the skin microbiome plays a potential role in regulating the skin symptoms in CKD patients.

It is reported that most (> 90%) of bacteria of the human skin microbiome are classified into four types: Actinobacteria, Firmicutes, Proteobacteria, and Bacteroidetes (Elizabeth and Julia, 2011). Indeed, the major bacterial phyla in our present study were also classified into the abovementioned four bacteria. Interestingly, a common feature of skin and gut microbial communities displayed in the present skin study and our previous gut study on CKD patients (Liu et al., 2021), such as Actinobacteria, exhibited higher abundance in both the skin and gut samples in patient group.

We noticed that Proteobacteria demonstrated slightly higher abundance in CKD patients than that in HC group. High level of Proteobacteria in the gut is responsible for unhealthy states, including CKD (Zhao et al., 2021). The enrichment of Proteobacteria in the skin is also reported to be associated with illnesses, such as leprosy and pustula (van Rensburg et al., 2015; Silva et al., 2018). In the present study, the high abundance of Proteobacteria in CKD group was ascribed to the high levels of *Ralstonia*, which accounted for 8.34% of the total abundance in CKD patients, and it was listed as the top 1 abundant bacterial genus in patients. The association between *Ralstonia* and health state has never been reported by human microbiome study. Further study is needed to explore its function in the skin.

The present study found that *Escherichia–Shigella* was not only increased in CKD patients but also can be listed as a moderate diagnostic biomarker in patients. *Escherichia–Shigella* was also considered a diagnostic tool in the gut of CKD patients in the study of Wu et al. (2020). Interestingly, our present study demonstrated that only CKD patients in Stage 1 had higher abundance of *Escherichia–Shigella* compared to HC group. Its abundance was not significantly correlated to patient’s eGFR. This finding was inconsistent to the study of Wu et al. (2020). In their study, *Escherichia–Shigella* in the gut was positively correlated to patient’s renal function (Wu et al., 2020). This inconsistence might be partially due to the different sample collection sites between the study of Wu et al. and our study. Although *Escherichia–Shigella* did not exhibit an association with eGFR, it was positively correlated to the levels of BUN, uric acid, and urine protein. As high concentrations of BUN, uric acid, and urine protein are also representative for the renal function in CKD patients (Vanholder et al., 2018), we can consider that high abundance of *Escherichia–Shigella* in patient’s skin is a sign of renal function damages.

Similar to the alteration characteristic of *Escherichia–Shigella* in the skin, the increasing *Akkermansia* can only be detected in CKD stage 1 patient compared to HC group. As we did not observe that there was a difference in the bacterial community between the groups of CKD stage 1 and HC, the alterations of *Escherichia–Shigella* and *Akkermansia* suggest that some individual bacteria change earlier than the alteration of the whole bacterial community, which might be useful for detecting early renal insufficiency. It is worthy to note that the observation of high abundance of *Akkermansia* in Stage 1 CKD patients rather than other stages indicates compensated mechanism in the skin, as the reduction in *Akkermansia* in the gut has been reported in psoriasis (Eppinga et al., 2016; Tan et al., 2018). It seems that *Oribacterium*, which is a strictly anaerobic bacterial genus (Sizova et al., 2014), plays an important role in regulating the skin symptoms and renal function in CKD patients, as it was negatively responding to pruritus severity and positively responding to the values of eGFR and 24-h urine volume. Until now, the function of *Oribacterium* in human microbiome has rarely been explored. Only a previous study reported the isolation of *Oribacterium* spp. from human subgingival dental plaque, and its major metabolic fermentation end products included acetate and lactate (Sizova et al., 2014). Acetate and lactate belong to short-chain fatty acids (SCFAs) (Koh et al., 2016). In human gut, SCFAs are the main energy source of colonocytes and crucial to gastrointestinal health (Canfora et al., 2015). Thus, it is worth to explore whether *Oribacterium* plays a probiotic role in the skin of CKD patients and how it regulates patients’ skin symptoms and renal function.

There are limitations that might generate bias in our present study that should be considered. On the one hand, the sample size was not comparative between the groups of patient and HC. CKD is prevalent in the elder population (McClure et al., 2017). For example, the average age of CKD patients in the present study was 59.32 years old. High prevalence of hypertension and diabetes is common in the elderly (Kalyani et al., 2017; Benetos et al., 2019). Over half of Chinese elderly people have hypertension (Hua et al., 2019), and almost 60% of elderly Chinese have diabetes (Zhao et al., 2016). To control the confounding factors caused by diabetes and hypertension, we had to exclude those with hypertension and/or diabetes at the recruitment of the present study. Therefore, it was hard to recruit healthy elderly who were free of diabetes and hypertension. On the other hand, we recruited the participants lasting for 2 years. We could not remove the confounding factor of temperature and humidity of the sample collection environment led by season changes.

In conclusion, our present study first found that the altered bacterial skin microbiome was associated with pruritus and renal functions in CKD patients. These findings might be useful for making skin probiotics supplements to relieve patients’ skin symptoms or renal function damage.

## Data Availability Statement

The datasets presented in this study can be found in online repositories. The names of the repository/repositories and accession number(s) can be found below: https://www.ncbi.nlm.nih.gov/, SRP370056.

## Ethics Statement

The studies involving human participants were reviewed and approved by the ethics committee of the Affiliated Wuxi Second Hospital of Nanjing Medical University (Ref. 2018051). The patients/participants provided their written informed consent to participate in this study. Written informed consent was obtained from the individual(s) for the publication of any potentially identifiable images or data included in this article.

## Author Contributions

Conceptualization: PJ, WG, and NF. Methodology: YT, YF, JS, LH, CG, PJ, and FY. Software: YG and JC. Validation: NF. Writing: YT and YG. Supervision: NF. Funding acquisition: NF. Project administration: LH, PJ, JC, JS, CG, YF, and FY. All authors contributed to the article and approved the submitted version.

## Funding

This study was supported by Wuxi “Taihu Talents Program” Medical and Health High-Level Talents Project (THRCJH20200901), Wuxi “Key Medical Discipline Construction” Municipal Clinical Medical Center (Municipal Public Health Center) Project (LCYXZX202103), Zhejiang Provincial Natural Science Foundation of China (LXR22H160001), and National Natural Science Foundation of China (81874142 and 82073041).

## Conflict of Interest

The handling editor JY declared a shared parent affiliation with the author YG at the time of review.

The remaining authors declare that the research was conducted in the absence of any commercial or financial relationships that could be construed as a potential conflict of interest.

## Publisher’s Note

All claims expressed in this article are solely those of the authors and do not necessarily represent those of their affiliated organizations, or those of the publisher, the editors and the reviewers. Any product that may be evaluated in this article, or claim that may be made by its manufacturer, is not guaranteed or endorsed by the publisher.

## References

[B1] Ahdoot R. S. Kalantar-Zadeh K. Burton J. O. Lockwood M. B. (2022). Novel Approach to Unpleasant Symptom Clusters Surrounding Pruritus in Patients With Chronic Kidney Disease and on Dialysis Therapy. Curr. Opin. Nephrol. Hypertens. 31, 63–71. doi: 10.1097/MNH.0000000000000752 34750335 PMC8633148

[B2] Benetos A. Petrovic M. Strandberg T. (2019). Hypertension Management in Older and Frail Older Patients. Circ. Res. 124, 1045–1060. doi: 10.1161/CIRCRESAHA.118.313236 30920928

[B3] Bjerre R. D. Bandier J. Skov L. Engstrand L. Johansen J. D. (2017). The Role of the Skin Microbiome in Atopic Dermatitis: A Systematic Review. Br. J. Dermatol. 177, 1272–1278. doi: 10.1111/bjd.15390 28207943

[B4] Canfora E. E. Jocken J. W. Blaak E. E. (2015). Short-Chain Fatty Acids in Control of Body Weight and Insulin Sensitivity. Nat. Rev. Endocrinol. 11, 577–591. doi: 10.1038/nrendo.2015.128 26260141

[B5] Collaboration GCKD (2020). Global, Regional, and National Burden of Chronic Kidney Disease, 1990-2017: A Systematic Analysis for the Global Burden of Disease Study 2017. Lancet 395, 709–733. doi: 10.1016/S0140-6736(20)30045-3 32061315 PMC7049905

[B6] Combs S. A. Teixeira J. P. Germain M. J. (2015). Pruritus in Kidney Disease. Semin. Nephrol. 35, 383–391. doi: 10.1016/j.semnephrol.2015.06.009 26355256 PMC5497472

[B7] Elizabeth A. G. Julia A. S. (2011). The Skin Microbiome. Nat. Rev. Microbiol. 9, 244–253. doi: 10.1038/nrmicro2537 21407241 PMC3535073

[B8] Eppinga H. Sperna W. C. Thio H. B. van der Woude C. J. Nijsten T. E. Peppelenbosch M. P. (2016). Similar Depletion of Protective Faecalibacterium Prausnitzii in Psoriasis and Inflammatory Bowel Disease, But Not in Hidradenitis Suppurativa. J. Crohns Colitis 10, 1067–1075. doi: 10.1093/ecco-jcc/jjw070 26971052

[B9] Erickson S. Kim B. S. (2019). Research Techniques Made Simple: Itch Measurement in Clinical Trials. J. Invest. Dermatol. 139, 264–269. doi: 10.1016/j.jid.2018.12.004 30665580 PMC8922716

[B10] Francuzik W. Franke K. Schumann R. R. Heine G. Worm M. (2018). Propionibacterium Acnes Abundance Correlates Inversely With Staphylococcus Aureus: Data From Atopic Dermatitis Skin Microbiome. Acta Derm. Venereol. 98, 490–495. doi: 10.2340/00015555-2896 29379979

[B11] Grice E. A. Kong H. H. Renaud G. Young A. C. Bouffard G. G. Blakesley R. W. (2008). A Diversity Profile of the Human Skin Microbiota. Genome Res. 18, 1043–1050. doi: 10.1101/gr.075549.107 18502944 PMC2493393

[B12] Hua Q. Fan L. Li J. (2019). 2019 Chinese Guideline for the Management of Hypertension in the Elderly. J. Geriatr. Cardiol. 16, 67–99. doi: 10.11909/j.issn.1671-5411.2019.02.001 30923539 PMC6431598

[B13] Huang C. Yi X. Long H. Zhang G. Wu H. Zhao M. (2020). Disordered Cutaneous Microbiota in Systemic Lupus Erythematosus. J. Autoimmun. 108, 102391. doi: 10.1016/j.jaut.2019.102391 31883828

[B14] Hu X. Ouyang S. Xie Y. Gong Z. Du J. (2020). Characterizing the Gut Microbiota in Patients With Chronic Kidney Disease. Postgrad. Med. 132, 495–505. doi: 10.1080/00325481.2020.1744335 32241215

[B15] Kalyani R. R. Golden S. H. Cefalu W. T. (2017). Diabetes and Aging: Unique Considerations and Goals of Care. Diabetes Care 40, 440–443. doi: 10.2337/dci17-0005 28325794 PMC5360288

[B16] Kim S. J. Zhang X. Cho S. B. Kim C. H. Park H. C. Moon S. J. (2021). Uremic Solutes of Indoxyl Sulfate and P-Cresol Enhance Protease-Activated Receptor-2 Expression *In Vitro* and *In Vivo* in Keratinocytes. Hum. Exp. Toxicol. 40, 113–123. doi: 10.1177/0960327120945758 32757783

[B17] Koh A. De Vadder F. Kovatcheva-Datchary P. Bäckhed F. Institute Of Medicine DOMA Wallenberg L. (2016). From Dietary Fiber to Host Physiology: Short-Chain Fatty Acids as Key Bacterial Metabolites. Cell 165, 1332–1345. doi: 10.1016/j.cell.2016.05.041 27259147

[B18] Liu F. Xu X. Chao L. Chen K. Shao A. Sun D. (2021). Alteration of the Gut Microbiome in Chronic Kidney Disease Patients and Its Association With Serum Free Immunoglobulin Light Chains. Front. Immunol. 12, 609700. doi: 10.3389/fimmu.2021.609700 33868230 PMC8047322

[B19] Mandrekar J. N. (2010). Receiver Operating Characteristic Curve in Diagnostic Test Assessment. J. Thorac. Oncol. 5, 1315–1316. doi: 10.1097/JTO.0b013e3181ec173d 20736804

[B20] Meijers B. Evenepoel P. Anders H. J. (2019). Intestinal Microbiome and Fitness in Kidney Disease. Nat. Rev. Nephrol. 15, 531–545. doi: 10.1038/s41581-019-0172-1 31243394

[B21] McClure M. Jorna T. Wilkinson L. Taylor J. (2017). Elderly Patients With Chronic Kidney Disease: Do They Really Need Referral to the Nephrology Clinic? Clin. Kidney J. 10, 698–702. doi: 10.1093/ckj/sfx034 28979782 PMC5622896

[B22] O'Neill A. M. Gallo R. L. (2018). Host-Microbiome Interactions and Recent Progress Into Understanding the Biology of Acne Vulgaris. Microbiome 6, 177. doi: 10.1186/s40168-018-0558-5 30285861 PMC6169095

[B23] Perez-Gomez M. V. Bartsch L. A. Castillo-Rodriguez E. Fernandez-Prado R. Fernandez-Fernandez B. Martin-Cleary C. (2019). Clarifying the Concept of Chronic Kidney Disease for non-Nephrologists. Clin. Kidney J. 12, 258–261. doi: 10.1093/ckj/sfz007 30976406 PMC6452188

[B24] Phan N. Q. Blome C. Fritz F. Gerss J. Reich A. Ebata T. (2012). Assessment of Pruritus Intensity: Prospective Study on Validity and Reliability of the Visual Analogue Scale, Numerical Rating Scale and Verbal Rating Scale in 471 Patients With Chronic Pruritus. Acta Derm. Venereol. 92, 502–507. doi: 10.2340/00015555-1246 22170091

[B25] Prevention CFDC (2021). Chronic Kidney Disease in the United States, 2021 (Atlanta, GA: US Department of Health and Human Services, Centers for Disease Control and Prevention).

[B26] Puneet A. Vinita G. Priyanka K. Jacek C. S. Stephan G. Mohamad G. (2021). Chronic Kidney Disease-Associated Pruritus. Toxins. 13(8):527. doi: 10.3390/toxins13080527. 34437400 PMC8402524

[B27] Ren Z. Fan Y. Li A. Shen Q. Wu J. Ren L. (2020). Alterations of the Human Gut Microbiome in Chronic Kidney Disease. Adv. Sci. (Weinh) 7, 2001936. doi: 10.1002/advs.202001936 33101877 PMC7578882

[B28] Silva P. Reis M. P. Avila M. P. Dias M. F. Costa P. S. Suhadolnik M. (2018). Insights Into the Skin Microbiome Dynamics of Leprosy Patients During Multi-Drug Therapy and in Healthy Individuals From Brazil. Sci. Rep. 8, 8783. doi: 10.1038/s41598-018-27074-0 29884862 PMC5993821

[B29] Sizova M. V. Muller P. A. Stancyk D. Panikov N. S. Mandalakis M. Hazen A. (2014). *Oribacterium Parvum* Sp. Nov. And *Oribacterium Asaccharolyticum* Sp. Nov., Obligately Anaerobic Bacteria From the Human Oral Cavity, and Emended Description of the Genus Oribacterium. Int. J. Syst. Evol. Microbiol. 64, 2642–2649. doi: 10.1099/ijs.0.060988-0. 24824639 PMC4129163

[B30] Stevens P. E. Levin A. (2013). Evaluation and Management of Chronic Kidney Disease: Synopsis of the Kidney Disease: Improving Global Outcomes 2012 Clinical Practice Guideline. Ann. Intern. Med. 158, 825–830. doi: 10.7326/0003-4819-158-11-201306040-00007 23732715

[B31] Tan L. Zhao S. Zhu W. Wu L. Li J. Shen M. (2018). The Akkermansia Muciniphila Is a Gut Microbiota Signature in Psoriasis. Exp. Dermatol. 27, 144–149. doi: 10.1111/exd.13463 29130553

[B32] Vanholder R. Gryp T. Glorieux G. (2018). Urea and Chronic Kidney Disease: The Comeback of the Century? (in Uraemia Research). Nephrol. Dial. Transplant. 33, 4–12. doi: 10.1093/ndt/gfx039 28407121

[B33] van Rensburg J. J. Lin H. Gao X. Toh E. Fortney K. R. Ellinger S. (2015). The Human Skin Microbiome Associates With the Outcome of and Is Influenced by Bacterial Infection. Mbio 6, e1315. doi: 10.1128/mBio.01315-15 PMC4600114 26374122

[B34] Wang X. Yang S. Li S. Zhao L. Hao Y. Qin J. (2020). Aberrant Gut Microbiota Alters Host Metabolome and Impacts Renal Failure in Humans and Rodents. Gut 69, 2131–2142. doi: 10.1136/gutjnl-2019-319766 32241904 PMC7677483

[B35] Wood D. Lachner N. Tan J. M. Tang S. Angel N. Laino A. (2018). A Natural History of Actinic Keratosis and Cutaneous Squamous Cell Carcinoma Microbiomes. MBIO 9(5):e01432–8. doi: 10.1128/mBio.01432-18 PMC6178618 30301852

[B36] Wu I. W. Lin C. Y. Chang L. C. Lee C. C. Chiu C. Y. Hsu H. J. (2020). Gut Microbiota as Diagnostic Tools for Mirroring Disease Progression and Circulating Nephrotoxin Levels in Chronic Kidney Disease: Discovery and Validation Study. Int. J. Biol. Sci. 16, 420–434. doi: 10.7150/ijbs.37421 32015679 PMC6990903

[B37] Zhao Y. Crimmins E. M. Hu P. Shen Y. Smith J. P. Strauss J. (2016). Prevalence, Diagnosis, and Management of Diabetes Mellitus Among Older Chinese: Results From the China Health and Retirement Longitudinal Study. Int. J. Public Health 61, 347–356. doi: 10.1007/s00038-015-0780-x 26755457 PMC4880519

[B38] Zhao J. Ning X. Liu B. Dong R. Bai M. Sun S. (2021). Specific Alterations in Gut Microbiota in Patients With Chronic Kidney Disease: An Updated Systematic Review. Ren. Fail 43, 102–112. doi: 10.1080/0886022X.2020.1864404 33406960 PMC7808321

