# Supplementary material for: Alteration of Skin Microbiome in CKD Patients Is Associated With Pruritus and Renal Function
Source: Front Cell Infect Microbiol. 2022 Jun 28;12:923581. doi: 10.3389/fcimb.2022.923581 (PMC9274276; doi:10.3389/fcimb.2022.923581)
Supplement: Supplementary file 1 [file Image_1.pdf]

(A)

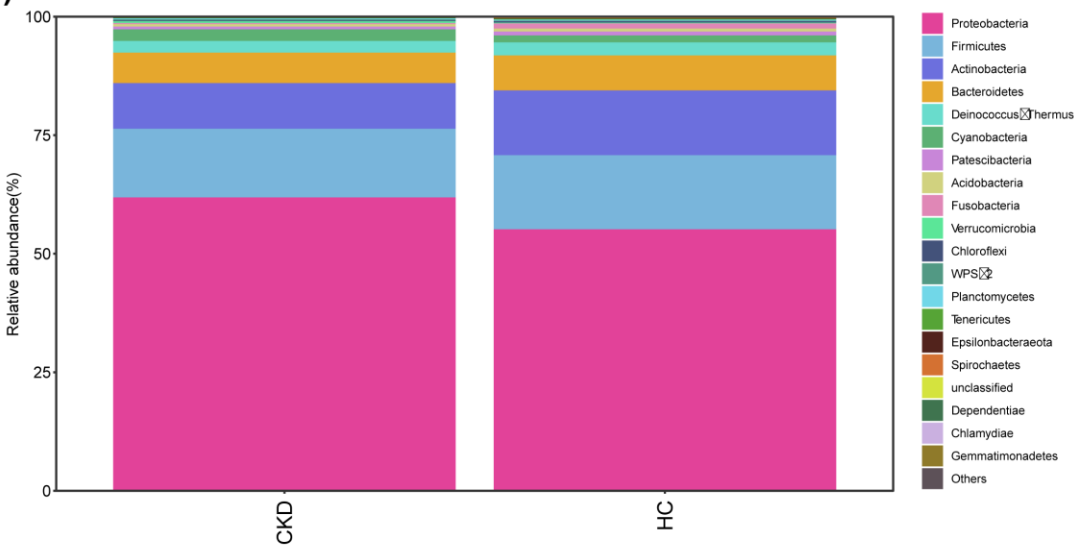

(B)

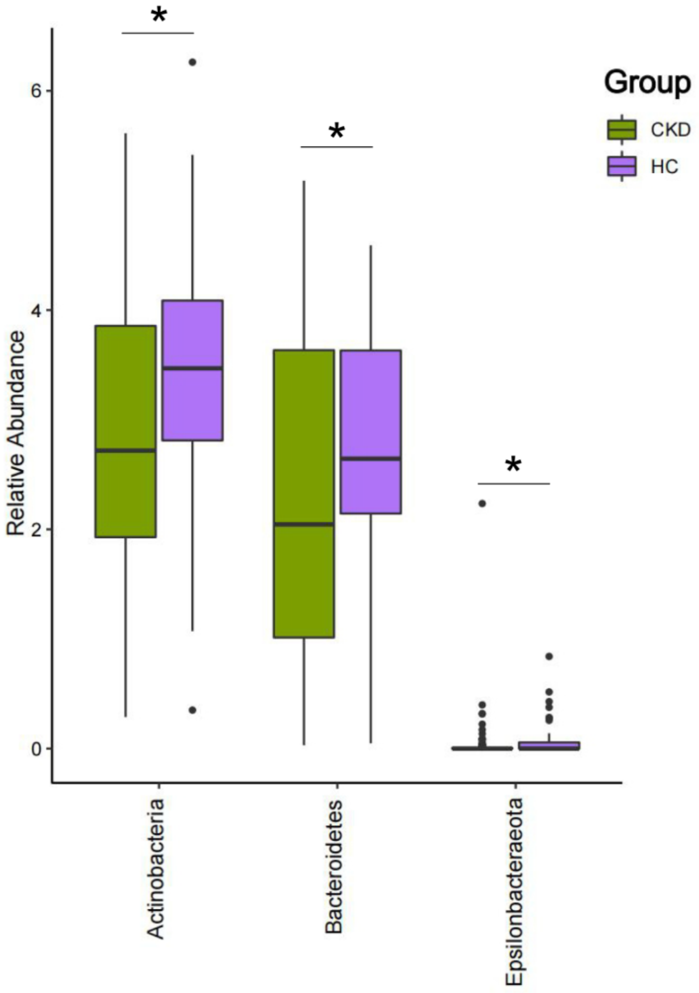

Figure S1 Bacterial phylum abundance and comparisons

(A) The top 20 most abundant bacterial phyla in the participants were displayed.

(B) Comparisons of the abundance of bacterial phylum between CKD and HC. Wilcoxon rank-sum test was performed and adjusted by Benjamini and Hochberg false discovery rate (FDR). * indicates $P<0.05$.
