# Supplementary material for: Alteration of Skin Microbiome in CKD Patients Is Associated With Pruritus and Renal Function
Source: Front Cell Infect Microbiol. 2022 Jun 28;12:923581. doi: 10.3389/fcimb.2022.923581 (PMC9274276; doi:10.3389/fcimb.2022.923581)
Supplement: Supplementary file 2 [file Table_1.doc]

**Table S1 Characteristics of p-CKD and nonP-CKD**

| **Parameters** | **P-CKD (n = 77)** | **nonP-CKD (n = 26)** | ***P* value** |
| --- | --- | --- | --- |
| Age, yr | 62.13 ± 16.11 | 51.00 ± 16.29 | 0.004 |
| Men | 40 (51.95) | 16 (61.54) | 0.396 |
| Body mass index (kg/m^2^) | 25.39 ± 6.07 | 25.56 ± 3.68 | 0.985 |
| eGFR (mL/min/1.73m^2^) | 52.27 ± 34.09 | 73.35 ± 36.59 | 0.009 |
| Serum urea (mmol/L) | 12.86± 11.15 | 9.06 ± 7.13 | 0.048 |
| Serum creatinine (mg/dL) | 224.71 ± 209.98 | 142.64 ± 123.31 | 0.019 |
| Serum uric acid | 399.73 ± 135.91 | 392.53 ± 145.93 | 0.819 |
| Urine protein | 1.66 ± 1.45 | 0.12 ± 0.60 | <0.001 |
| 24-hour urine protein | 2911.87 ± 2152.90 | 3039.92 ± 2553.94 | 0.814 |
| 24-hour urine volume | 1812.01 ± 6199.99 | 2070.45 ± 642.48 | 0.103 |
| Urine microalbumin | 179.18 ± 56.15 | 174.88 ± 56.89 | 0.793 |
| Urine albumin/creatine ratio | 39.81 ± 13.98 | 28.76 ± 20.16 | 0.013 |

Pearson’s Chi-square/Fisher’s exact test was used to compare dichotomous variables, and an independent *t*-test was used to compare continuous variables.
